# Pre-procedural renal resistive index accurately predicts contrast-induced acute kidney injury in patients with preserved renal function submitted to coronary angiography

**DOI:** 10.1007/s10554-016-1039-1

**Published:** 2016-12-19

**Authors:** Maciej T. Wybraniec, Maria Bożentowicz-Wikarek, Jerzy Chudek, Katarzyna Mizia-Stec

**Affiliations:** 1 First Department of Cardiology, School of Medicine in Katowice, Medical University of Silesia, 47 Ziołowa St., 40-635 Katowice, Poland; 2 Upper Silesia Medical Centre, Public Hospital No. 7, Katowice, Poland; 3 Department of Pathophysiology, Medical University of Silesia, Katowice, Poland

**Keywords:** Contrast-induced acute kidney injury, CI-AKI, Renal resistive index, RRI, Renal pulsatility index, RPI

## Abstract

The study aimed to evaluate the clinical utility of ultrasonographic intra-renal blood flow parameters, together with the wide range of different risk factors, for the prediction of contrast-induced acute kidney injury (CI-AKI) in patients with preserved renal function, referred for coronary angiography or percutaneous coronary interventions (CA/PCI). This prospective study covered 95 consecutive patients (69.5% men; median age 65 years) subject to elective or urgent CA/PCI. Data regarding 128 peri-procedural variables were collected. Ultrasonographic intra-renal blood flow parameters, including renal resistive index (RRI) and pulsatility index (RPI), were acquired directly before the procedure. CI-AKI was defined as ≥50% relative or ≥0.3 mg/dL absolute increase of serum creatinine 48 h after procedure. CI-AKI was confirmed in nine patients (9.5%). Patients with CI-AKI had higher SYNTAX score (p = 0.0002), higher rate of left main disease (p < 0.00001), peripheral artery disease (PAD; p = 0.02), coronary artery anomaly (p = 0.017), more frequently underwent surgical revascularization (p = 0.0003), ‘had greater...’ intima-(p = 0.004) and extra-medial thickness (p = 0.001), and received higher contrast media dose (p = 0.049), more often overused non-steroidal anti-inflammatory drugs (p = 0.001), and had substantially higher pre-procedural RRI (0.69 vs. 0.62; p = 0.005) and RPI values (1.54 vs. 1.36; p = 0.017). Logistic regression confirmed age, SYNTAX score, presence of PAD, diabetes mellitus, and pre-procedural RRI independently predicted CI-AKI onset (AUC = 0.95; p < 0.0001). Pre-procedural RRI > 0.69 had 78% sensitivity and 81% specificity in CI-AKI prediction. High pre-procedural RRI seems to be a useful novel risk factor for CI-AKI in patients with preserved renal function. Coronary, peripheral and renal vascular pathology contribute to the development of CI-AKI following CA/PCI.

## Introduction

Ubiquitous application of contrast media (CM) confers a major risk of contrast induced-acute kidney injury (CI-AKI). The rate of this frequently overlooked complication varies from 2.5 to 13.1% of coronary angiographies and percutaneous coronary interventions (CA/PCI) [1–3] and has been repeatedly linked to increased morbidity and mortality [1, 4]. Given the late serum creatinine concentration (SCr) surge following renal injury and so far unsuccessful search for early diagnostic markers of CI-AKI [5], adequate pre-procedural risk stratification and prevention play strategic role in decreasing the burden of CI-AKI. A wide range of CI-AKI risk factors has so far been established, such as impaired baseline kidney function, volume of CM applied, dehydration, advanced age, diabetes mellitus and atherosclerosis severity [1–3, 6]. The development of renal injury is believed to be triggered by high osmolality and viscosity of CM, leading to increased renal vascular resistance [7] and renal tubular hypoxia, eventually causing tubular cell apoptosis [8]. Preexisting increased vascular stiffness, reflected by high central arterial pulse pressure could facilitate CI-AKI via impaired renal blood flow auto-regulation [9]. The Doppler ultrasound of interlobular and/or arcuate arteries delivers indirect insight into renal hemodynamics. Out of all renal blood flow parameters, only renal resistive index (RRI) has been shown to be clinically useful, primarily in the evaluation of renovascular hypertension [10] or the kidney allograft function [11] or the risk of acute kidney injury (AKI) persistence [12]. Of note, RRI assessed in the setting of intensive care unit (ICU) accurately predicted the development and persistence of AKI [13]. RRI could represent an indicator of diffuse arterial stiffness and cardiovascular risk factor [14], predicting long-term morbidity and mortality [15]. Yet, several factors can interfere with RRI values, including pulse blood pressure, heart rate and rhythm, presence of significant aortic valve stenosis, age, renal interstitial disease or vascular compliance [16]. Also, patients with chronic kidney disease were shown to have an altered and delayed vascular response to contrast media [7].

We thus hypothesized that RRI, after adjustment for its covariates, could serve as an indicator of baseline renal vascular resistance and contribute to CI-AKI risk stratification in patients devoid of preexisting kidney pathology. Accordingly, the purpose of the study was to evaluate the clinical significance of preoperative ultrasonographic parameters of intra-renal blood flow, along with numerous pre- and intra-operative risk factors, for the prediction of CI-AKI in patients with coronary artery disease (CAD) and preserved renal function, referred for elective and urgent coronary angiography.

## Methods

This research represents a prospective observational study, which involved 95 consecutive patients with either stable angina (SA) or non-ST-elevation acute coronary syndrome (NSTE-ACS). All patients were hospitalized in the department of cardiology between 2013 and 2015 and referred for planned or urgent CA/PCI. Patients were enrolled for the study during the initial check-up at hospital, prior to the procedure. The study was conducted in adherence to Declaration of Helsinki Guidelines and was approved by the local Ethics Committee. On admission all patients gave their written informed consent to participation in the research.

Inclusion criteria comprised: (a) stable angina with high pre-test probability of CAD or SA with positive treadmill electrocardiographic stress test or echocardiographic dobutamine test; or (b) non-ST-elevation acute coronary syndrome that met diagnostic criteria as established in ESC NSTE-ACS 2011 guidelines.

Exclusion criteria were as follows: (a) significant hemodynamic instability (cardiogenic shock; Killip class >2; systolic blood pressure (SBP) <90 mmHg or acute decrease by 40 mmHg or catecholamine use dependence); (b) acute or chronic respiratory failure (blood oxygen saturation <90%); (c) severe heart failure with left ventricular ejection fraction (LVEF) <35%; (d) chronic kidney disease with eGFR < 50 mL/min/1.73 m^2^ or proteinuria >500 mg/L; (e) active urinary tract infection; (f) evidence of renal artery stenosis or hydronephrosis (g) moderate to severe aortic valve stenosis; (h) severe valvular heart disease of any kind; (i) high pulse pressure >80 mmHg; (j) tachycardia >100 bpm or bradycardia <50 bpm; (k) severe obesity (body mass index, BMI > 40 kg/m^2^); (l) active neoplastic disease; (m) liver dysfunction (any hepatic aminotransferase >3× upper reference limit); (n) intolerance of statin or history of rhabdomyolysis or myositis or (o) age <18 or >80 years old.

The primary endpoint was the onset of CI-AKI defined according to Acute Kidney Injury Network criteria, namely ≥50% relative or ≥0.3 mg/dL absolute increase of serum creatinine concentration (SCr) at 48 h after procedure [17]. Data regarding 128 pre- and intra-procedural variables were collected in all study participants and compared between CI-AKI and non-CI-AKI group. Patients were meticulously interviewed and former discharge summaries were revised in order to collect demographic and comorbidities data. The list of all current medications was obtained. Routine laboratory tests and anthropometric measurements, and baseline echocardiographic study and carotid artery scans were performed on admission in all study participants.

Blood samples were collected from antecubital or radial vein at admission, 24 and 48 h following CA. Baseline standard laboratory panel, including serum creatinine concentration (SCr) was evaluated at admission, while 24-h and 48-h blood samples were assayed only for SCr.

Ultrasonographic indices of intra-renal blood flow in arcuate/interlobular arteries were assessed directly before and 1 h after the procedure. The renal Doppler ultrasound was performed by one experienced physician using Vivid 7 (GE Healthcare) with a 5C probe (4.4–6.7 MHz) designed for high-frequency vascular applications within abdominal cavity. The study was performed on patients in supine position, after 10 min of rest and prior blood pressure measurement. Initially, length and width of renal artery was determined and main renal artery stenosis was excluded by means of peak systolic and end-diastolic flow velocity calculation.

Subsequently, ultrasonographic parameters of renal blood flow in arcuate/interlobular arteries, located on the border of renal cortex and core were evaluated using a 2–4 mm pulse-wave Doppler gate. Intra-renal blood flow velocity was assessed using the smallest scale allowing for precise measurements without the aliasing phenomenon (pulse repetition frequency), but with the highest possible gain and the lowest possible value of wall motion filter. The Doppler intra-renal blood flow parameters comprised peak systolic (PSV) and end-diastolic velocity (EDV), augmentation index (AI), acceleration time (AT), and further derivatives, including renal resistive index (RRI) and pulsatility index (RPI). RRI was defined as RRI = (PSV − EDV)/PSV, while RPI was defined as: RPI = (PSV − EDV)/MV, where MV stands for mean flow velocity. Renal to abdominal aorta flow velocity ratio (RAR) was calculated as the ratio of arithmetic mean of PSV in main renal arteries and PSV in abdominal aorta. The measurements were performed three times for each kidney during pre-procedural evaluation on different intra-renal arteries (upper, mid and lower lobe of kidney). The final result represented an arithmetic mean of three blood flow scans of right and left kidney (six measurements for pre-procedural evaluation). The intra-observer variability was 96% for RRI and 91% for RPI. The duration of the bilateral renal Doppler ultrasound was approximately 5–10 min.

Echocardiographic check-up was conducted using Vivid 7 (GE Healthcare) with a 2.5-MHz probe by a single investigator. Subsequently, carotid scans were performed using a Logic 7, GE ultrasound machine with a 7–12-MHz linear array transducer and then analyzed using quantitative analysis package (Siemens) characterized by an axial resolution of 0.001 mm.

All the study participants received standard prophylaxis of CI-AKI. Only low-osmolar and iso-osmolar CM at the lowest possible dose was used in all study participants. CA and PCI were performed by five different experienced interventional cardiologists. All the patients with moderately impaired kidney function (eGFR 50–60 mL/min) received an IV infusion of 0.9% saline at a rate of 1 mL/kg/h starting from 12 h before to 24 after CA. Patients with eGFR > 60 mL/min only obtained a single infusion of 500 mL 0.9% saline prior to procedure.

HTN was diagnosed if blood pressure exceeded 140/90 mmHg on two separate measurements during index hospitalization or in the event of former HTN diagnosis or antihypertensive medication use. Diabetes mellitus /impaired fasting glucose/impaired glucose tolerance were analyzed jointly. DM was diagnosed if fasting blood glucose was >125 mg/dL on two separate days or if patients received insulin or oral hypoglycemic agents. Non-steroidal anti-inflammatory drugs (NSAID) use was defined >1 dose/per week use of NSAIDs. The pre-procedural risk of CI-AKI development was additionally estimated based on Mehran et al. risk score [2]. Hypotension during procedure was defined as the sudden decrease of invasively monitored SBP below 90 mmHg or by more than 40 mmHg.

The coronary anatomy and morphology of coronary lesions were assessed by an experienced interventional cardiologist. The SYNTAX score was calculated using widely accessible online calculator.

Statistical analysis was performed using MedCalc v.14.8.1 software (MedCalc Software, Ostend, Belgium). Quantitative variables were presented as the mean value ± standard deviation (SD) or median (boundaries of 25 and 75 percentile) and qualitative parameters were expressed as number and percentage. The type of distribution was verified using Shapiro–Wilk test. In case of normally-distributed variables, Student’s *t* test for unpaired samples was used, while Mann–Whitney *U* test was implemented in non-normally distributed parameters. Qualitative variables were compared using the Pearson’s Chi square test. Initially all CI-AKI predictor variables were evaluated in univariate analysis and odds ratios (OR) with 95% confidence interval (CI) were calculated. All the variables with p < 0.1 in univariate model were incorporated into the logistic regression analysis model. The area under (AUC) receiver operating characteristic (ROC) curve for the model was calculated. Optimum cut-off point of pre-procedural renal blood flow parameters were established using Youden’s J statistic estimation. In order to determine the relationship between variables, the Pearson’s and Spearman coefficient of correlation were calculated. A ‘p’ value of less than 0.05 was regarded as statistically significant.

## Results

The study involved 95 consecutive patients referred for elective or urgent coronary angiography. Demographic and clinical characteristics are highlighted in Table 1. No gender-based differences were observed. The majority of study participants were diagnosed with non-ST-elevation acute coronary syndrome (n = 54, 56.8%) and stable angina was slightly less frequent (n = 41; 43.2%). The vast majority of study participants received angiotensin-converting enzyme inhibitors (n = 84, 88.4%), beta-blockers (n = 80, 84.2%), statins (n = 84, 88.4%). Considerably smaller proportion of patients was treated with calcium channel blockers (n = 26, 27.4%), mineralocorticoid receptor antagonists (n = 16, 16.8%), loop or thiazide diuretics (n = 28, 29.5%), nitrates (n = 19, 20%), metformin (n = 16, 16.8%), trimetazidine (n = 11, 11.6%) and allopurinol (n = 7, 7.4%). Thirteen patients (13.7%) overused non-steroidal anti-inflammatory drugs. The median hospitalization time was 4 (3; 4) days. The general Doppler parameters of renal and intra-renal blood flow are presented in Table 2.

**Table 1:** Demographic and clinical characteristics of the study population

Variable	N = 95 absolute count (%) or median (25–75 percentile) or mean ± SD
Age (years)	65 (59; 71)
Men	66 (69.5%)
Body mass index (kg/m^2^)	28.4 (25.9; 32.3)
Obesity	39 (41.1%)
Waist-to-hip ratio	1.01 (0.96; 1.08)
Cigarette smoking	58 (61.1%)
Acute coronary syndrome	54 (56.3%)
Non-ST-elevation myocardial infarction	28 (29.5%)
Mehran’s CI-AKI risk (%)	7.5 (7.5; 14.0)
SYNTAX score (pts)	12 (4; 25)
Left main disease	10 (10.5%)
Arterial hypertension	91 (95.8%)
DM/IFG/IGT	37 (38.5%)
Dyslipidemia	88 (92.6%)
Atrial fibrillation (paroxysmal/persistent)	21 (22.1%)
Peripheral artery disease	16 (16.8%)
Chronic obstructive pulmonary disease	9 (9.5%)
History of myocardial infarction	41 (43.2%)
History of stroke/transient ischemic attack	7 (7.4%)
Heart rate (bpm)	64 (57; 75)
Systolic blood pressure (mmHg)	135 (120; 145)
Diastolic blood pressure (mmHg)	80 (70; 90)
Pulse blood pressure (mmHg)	60 (50; 70)
Left ventricular ejection fraction (%)	55 (50; 60)
E/e′	8.6 (6.7; 12.1)
Mitral valve insufficiency (mild-moderate)	61 (63.5%)
Left atrium diameter (mm)	40 (36; 43)
Left ventricular mass index (g/m^2^)	102 (90; 119)
Intima-media thickness (mm)	0.09 ± 0.03
Extra-medial thickness (mm)	0.07 (0.05; 0.08)
High sensitivity troponin T (ng/mL)^a^	0.02 (0.01; 0.03)
Hemoglobin (g/dL)	13.9 ± 1.2
White blood cell count (1000/mm^3^)	6.9 (5.7; 7.9)
Platelet count (1000/mm^3^)	197 (175; 253)
Alanine transferase (IU/L)	20 (16; 29)
Fasting glucose (mg/dL)	95 (88; 111)
Total cholesterol (mg/dL)	152.7 ± 29.1
Urine specific gravity	1.02 (1.01; 1.02)
SCr-baseline (mg/dL)	0.93 (0.79; 1.13)
Baseline eGFR by MDRD (mL/min/1.73 m^2^)	80.7 ± 20.8

*CI-AKI* contrast-induced acute kidney injury, *DM/IFG/IGT* diabetes mellitus/impaired fasting glucose/impaired glucose tolerance, *SCr* serum creatinine concentration, *eGFR* estimated glomerular filtration rate

^a^Patients with acute coronary syndrome only

**Table 2:** Pre-procedural renal Doppler ultrasound

Variable	N = 95 absolute count (%) or median (25–75 percentile) or mean ± SD
Abdominal Ao V_max_ (m/s)	0.59 ± 0.13
PSV—renal artery (m/s)	0.64 ± 0.12
EDV— renal artery (m/s)	0.26 (0.23; 0.29)
RAR	1.06 (0.91; 1.23)
PSV—intra-renal (m/s)	0.42 ± 0.096
EDV—intra-renal (m/s)	0.16 ± 0.05
Renal resistive index	0.63 ± 0.07
Renal pulsatility index	1.38 ± 0.22
Renal AT (m/s)	59.0 (51.0; 70.0)
Renal AI (m/s^2^)	4.1 (3.6; 4.6)

*PSV* peak systolic velocity, *EDV* end-diastolic velocity, *AT* acceleration time, *AI* acceleration index, *Ao* aorta, *V*_*max*_ maximal velocity, *RAR* renal-aortic flow velocity index

Following coronary angiography, 44.2% (n = 42) of patients were referred for direct PCI, while 14.7% (n = 14) required elective or urgent coronary artery bypass grafting (CABG). Operators predominantly used femoral access, while radial approach was chosen only in 18.8% of cases (n = 18). The median duration of the procedure was 36 min. (25; 50). Drug-eluting stents were used exclusively in all study participants qualified for PCI. No patients required intra-aortic balloon pump use during the peri- and post-procedural period. Transient period of intra-procedural hypotension occurred in five patients (5.3%). Fractional flow reserve and intravascular ultrasound were utilized in only one patient respectively (1.1%).

During the procedure merely low-osmolar (iopromide or iomeprol; n = 84, 88.4%) or iso-osmolar CM (iodixanol; n = 10, 10.53%) were utilized. The median volume of administered CM was 100 mL (80; 180). The volume of CM to weight ratio was equal to 1.27 mL/kg (0.85; 2.25), and the volume adjusted to creatinine clearance was 1.47 (0.82; 2.20).

The CI-AKI defined by AKI Network criteria occurred in nine patients (9.5%). The median SCr at 24 h after CA/PCI was 0.96 (0.79; 1.17) mg/dL, while at 48 h SCr amounted to 1.01 (0.81; 1.20) mg/dL. Seven patients suffered from mild stage 1 AKI, defined by relative 1.5-2-fold SCr increase, whereas two subjects exhibited more severe AKI at stage 2 with 2-3-fold relative SCr increase. None of the study participants required dialysis therapy. Local vascular complications were reported in 11 patients (11.6%). No deaths occurred during the index hospitalization.

Data regarding inter-group differences of qualitative and quantitative parameters are denoted in Tables 3 and 4 respectively. Patients with CI-AKI were characterized by substantially higher pre-procedural RRI (0.69 vs. 0.62; p = 0.005) and RPI values (1.54 vs. 1.36; p = 0.017). There was a trend towards lower intra-renal EDV in patients with onset of CI-AKI (0.13 ± 0.04 vs. 0.16 ± 0.05 m/s, p = 0.089), while intra-renal PSV was almost identical in both groups (0.42 ± 0.1 vs. 0.42 ± 0.1; p = 0.98). Intra-renal AT and AI, as well as RAR and main renal artery PSV and EDV did not differ between CI-AKI and non-CI-AKI group (Table 4).

**Table 3:** Comparison of CI-AKI and non-CI-AKI group in terms of different qualitative risk factors

Variable	N = 86 CI-AKI AKIN (−) n (%)	N = 9 CI-AKI AKIN (+) n (%)	p*
Men	58 (67.4%)	8 (88.9%)	0.184
Obesity	35 (40.7%)	4 (44.4%)	0.828
Cigarette smoking	52 (60.5%)	6 (66.7%)	0.716
hsTnT—positive	25 (29.1%)	3 (33.3%)	0.347
ACS (UA or NSTEMI)	47 (54.7%)	7 (77.8%)	0.183
History of acute kidney injury	4 (4.7%)	1 (11.1%)	0.398
History of myocardial infarction	35 (40.7%)	6 (66.7%)	0.135
Peripheral artery disease	12 (13.9%)	4 (44.4%)	0.020
Arterial hypertension	82 (95.3%)	9 (100.0%)	0.509
Atrial fibrillation	20 (23.3%)	1 (11.1%)	0.403
Dyslipidemia	79 (91.9%)	9 (100%)	0.374
DM/IFG/IGT	31 (36.0%)	6 (66.7%)	0.073
Mild proteinuria	8 (9.4%)	1 (11.1%)	0.869
Chronic obstructive pulmonary disease	8 (9.3%)	1 (11.1%)	0.860
Radial vascular access	17 (19.8%)	1 (11.1%)	0.528
Coronary artery anomaly	3 (3.5%)	2 (22.2%)	0.017
Left main disease	5 (5.8%)	5 (55.6%)	<0.0001
SYNTAX score >32 pts	4 (4.7%)	6 (66.7%)	<0.0001
Referral for CABG	9 (10.5%)	5 (55.6%)	0.0003
Iso-osmolar contrast media	8 (9.3%)	2 (22.2%)	0.229
Time of procedure: 6 am—noon	43 (50.0%)	1 (11.1%)	0.026
Time of procedure: noon—6 pm	30 (34.9%)	6 (66.7%)	0.062
PCI ad hoc	38 (44.2%)	4 (44.4%)	0.988
Hypotension during procedure	4 (4.6%)	1 (11.1%)	0.409
Mitral valve insufficiency (mild–moderate)	56 (65.1%)	5 (55.6%)	0.569
Regional wall motion abnormalities	45 (52.3%)	7 (77.8%)	0.144
LVEF < 50%	21 (24.4%)	3 (33.3%)	0.558
E/e′ ≥ 12	20 (23.3%)	2 (22.2%)	0.930
Statin therapy prior to contrast exposure	75 (87.2%)	9 (100.0%)	0.254
NSAIDs	8 (9.3%)	5 (55.6%)	0.001

*ACS* acute coronary syndrome, *UA* unstable angina, *NSTEMI* non-ST-elevation myocardial infarction, *IFG* impaired fasting glucose, *IGT* impaired glucose tolerance, *PCI* percutaneous coronary intervention, *NSAID* non-steroidal anti-inflammatory drug, *ACEI/ARB* angiotensin converting enzyme inhibitor/angiotensin receptor blocker, *LVEF* left ventricular ejection fraction

*Pearson’s Chi^2^ test

**Table 4:** Difference between CI-AKI and non-CI-AKI group in terms of different quantitative parameters

Variable	CI-AKI (−); N = 86 median (25–75 percentile) or mean ± SD	CI-AKI (+); N = 9 median (25–75 percentile) or mean ± SD	p
Age (years)	65 (57; 70)	69 (62; 74)	0.108^a^
BMI (kg/m^2^)	28.4 (26.4; 32.0)	24.5 (24.1; 33.2)	0.381^a^
Pulse BP (mmHg)	60 (50; 70)	50 (40; 70)	0.492^a^
SYNTAX score (pts)	11 (4; 22)	36 (25; 42)	0.0002^a^
Volume of contrast (mL)	100 (70; 160)	120 (100; 300)	0.049^a^
Volume of contrast/weight (mL/kg)	1.20 (0.81; 2.11)	1.43 (1.28; 4.11)	0.031^a^
LVEF (%)	55 (50; 60)	50 (50; 55)	0.297^a^
E/e′	8.6 (6.7; 12.1)	7.1 (5.7; 8.9)	0.389^a^
LVMI (g/m^2^)	102 (91; 120)	99 (86; 112)	0.425^a^
IMT (mm)	0.09 ± 0.026	0.12 ± 0.04	0.004^b^
EMT (mm)	0.06 (0.05; 0.08)	0.09 (0.08; 0.10)	0.001^a^
hsTnT (ng/mL)	0.02 (0.01; 0.04)	0.01 (0.01; 0.03)	0.549^a^
Hemoglobin (g/dL)	13.96 ± 1.192	13.30 ± 1.48	0.123^b^
ALT (IU/L)	20 (16; 29)	16 (13; 22)	0.051^a^
Fasting glucose (mg/dL)	95 (86; 111)	105 (95; 111)	0.161^a^
Baseline SCr (mg/dL)	0.93 (0.79; 1.12)	1.02 (0.80; 1.13)	0.879^a^
Baseline eGFR by MDRD (mL/min/m^2^)	80.32 ± 19.99	83.79 ± 29.08	0.637^b^
QTc interval (ms)	420 (400; 440)	440 (427.5; 455)	0.087^a^
Abdominal Ao Vmax (m/s)	0.60 ± 0.13	0.52 ± 0.15	0.108^b^
PSV renal artery (m/s)	0.64 ± 0.12	0.62 ± 0.10	0.667^b^
EDV renal artery (m/s)	0.26 (0.23; 0.30)	0.28 (0.23; 0.30)	0.763^a^
RAR	1.06 (0.91; 1.29)	1.09 (1.02; 1.54)	0.239^a^
PSV intra-renal (m/s)	0.42 ± 0.10	0.42 ± 0.09	0.984^b^
EDV intra-renal (m/s)	0.16 ± 0.05	0.13 ± 0.04	0.089^b^
RRI	0.62 ± 0.06	0.69 ± 0.083	0.005^b^
RPI	1.37 (1.24; 1.49)	1.52 (1.48; 1.63)	0.017^a^
Renal AT (ms)	57.3 (50.5; 70.0)	68.0 (61.0; 69.5)	0.384^a^
Renal AI (m/s^2^)	4.10 (3.60; 4.60)	4.05 (3.85; 4.85)	0.712^a^

*BMI* body mass index, *PSV* peak systolic velocity, *EDV* end-diastolic velocity, *AT* acceleration time, *AI* acceleration index, *Ao* aorta, *V*_*max*_ maximal velocity, *RAR* renal-aortic flow velocity index, *ALT* alanine tranferase, *LVEF* left ventricular ejection fraction, *PWT* posterior wall thickness, *IMT* intima-media thickness, *EMT* extra-medial thickness, *hsTnT* high-sensitivity troponin T, *TDI* tissue Doppler imaging

^a^Mann–Whitney *U* test

^b^Student’s *t* test

**Fig. 1:**
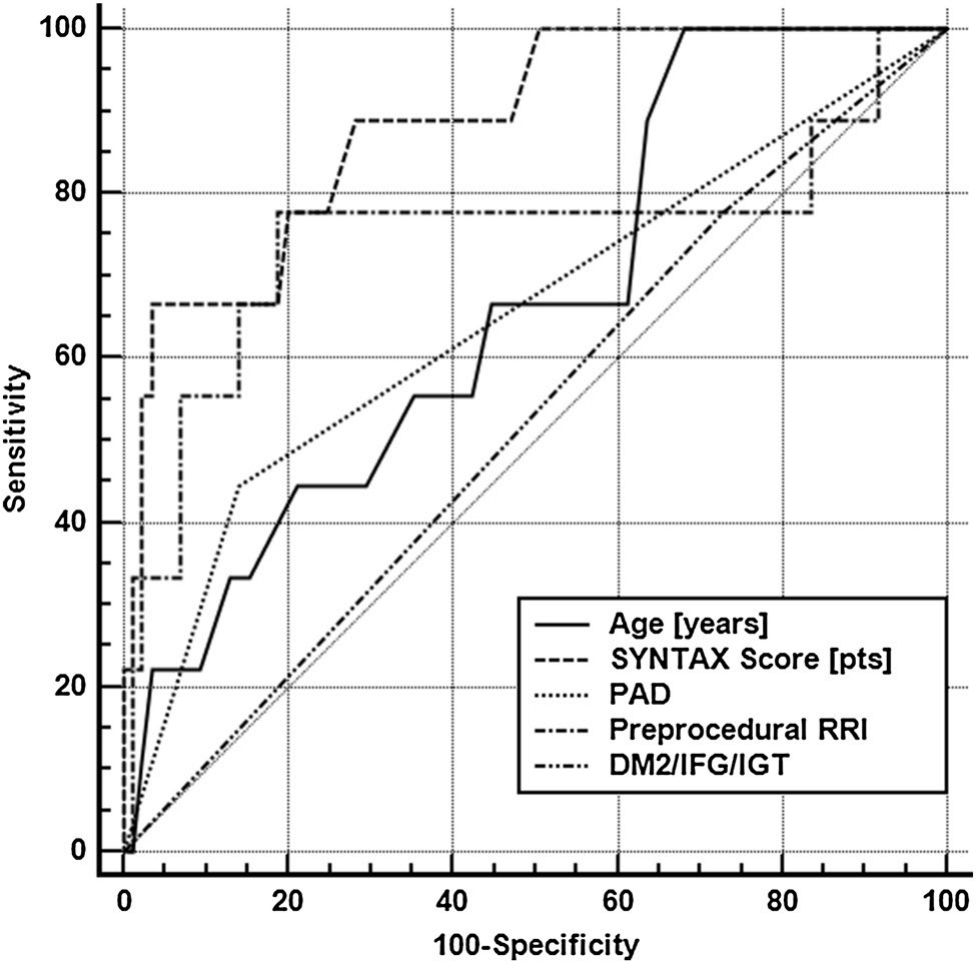
Receiver operator characteristic curve of the independent predictors of contrast-induced acute kidney injury onset. *DM* diabetes mellitus, *IFG* impaired fasting glucose, *IGT* impaired glucose tolerance, *RRI* renal resistive index, *PAD* peripheral artery disease

The results of the univariate analysis are presented in Table 5. Logistic regression analysis confirmed that SYNTAX score (OR = 1.19 per 1 pt, 95% CI 1.061–1.351, p = 0.0035) and pre-procedural RRI value (OR = 1.26 per 0.01, 95% CI 1.036–1.545, p = 0.021) were the strongest independent predictors of CI-AKI development. In addition, older age (OR = 1.19 per 1 year; 95% CI 1.0002–1.433, p = 0.049), the presence of peripheral artery disease (PAD; OR = 14.3, 95% CI 1.1–230.6, p = 0.045) and diabetes mellitus (OR = 20.5; 95% CI 1.01–600.54, p = 0.048) also heralded the onset of CI-AKI. The area under the receiver operating characteristic curve (Fig. 1) for the model was 0.95 (95% CI 0.88–0.98; p < 0.0001; Hosmer–Lemeshow goodness of fit p = 0.51).

**Table 5:** Univariate analysis of different pre- and peri-procedural risk factors of CI-AKI

Variable	OR	95% CI	p
Age (per 1 year)	1.09	0.99–1.20	0.091
Acute coronary syndrome	2.91	0.56–15.11	0.199
Hospitalization time (per 1 day)	1.37	0.97–1.94	0.076
Peripheral artery disease	4.93	1.14–21.42	0.033
Diabetes mellitus/IFG/IGT	3.55	0.81–15.48	0.091
Coronary artery anomaly	7.90	1.10–56.91	0.040
Left main disease	20.25	4.02–101.94	0.0002
SYNTAX score (per 1 pt)	1.14	1.05–1.22	0.001
SYNTAX score >32 pts	40.50	7.15–229.41	<0.0001
Referral for CABG	10.69	2.38–48.15	0.002
Volume of contrast (per 10 mL)	1.08	1.01–1.16	0.024
Volume of contrast to weight ratio	1.99	1.11–3.60	0.022
Intima media thickness (per 0.1 mm)	1.45	1.10–1.92	0.010
Extra-media thickness (per 0.1 mm)	1.92	1.27–2.90	0.002
NSAIDs use	12.19	2.66–55.86	0.001
PSV renal artery (per 1 m/s)	0.26	0.0006–118.81	0.664
EDV renal artery (per 1 m/s)	0.48	0.001–159.16	0.801
RAR	9.72	1.01–93.26	0.049
PSV—intra-renal (per 1 m/s)	0.93	0.0007–1324.98	0.983
EDV—intra-renal (per 1 m/s)	<0.001	<0.00001–10.75	0.095
Renal AT (per 1 ms)	0.99	0.96–1.03	0.773
Renal AI (per 1 m/s^2^)	1.22	0.68–2.20	0.499
RRI (per 0.01)	1.16	1.03–1.29	0.011
RPI (per 0.01)	1.04	1.00–1.07	0.028

*IFG* impaired fasting glucose, *IGT* impaired glucose tolerance, SBP systolic blood pressure, *DBP* diastolic blood pressure, *RAR* renal-aortic flow velocity index, *PSV* peak systolic velocity, *EDV* end-diastolic velocity, *AT* acceleration time, *AI* acceleration index, *Ao* aorta, *V*_*max*_ maximal velocity, *RAR* renal-aortic flow velocity index, *TDI* tissue doppler imaging, *NSAID* non-steroidal anti-inflammatory drugs

**Fig. 2:**
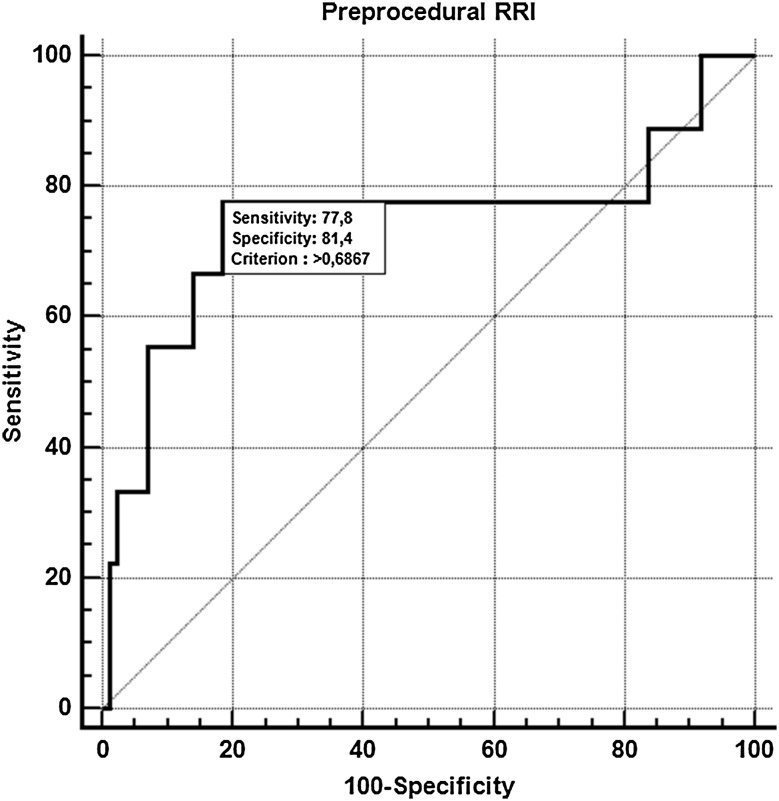
Receiver operator characteristic curve and threshold for contrast-induced acute kidney injury prediction of pre-procedural renal resistive index. *RRI* renal resistive index

The ROC curve analysis revealed that pre-procedural RRI had high predictive power for CI-AKI prediction (AUC = 0.75, 95% CI 0.65–0.83; p = 0.03). The threshold value of RRI > 0.69, associated with the calculated Youden’s J statistic, had sensitivity of 78%, specificity of 81%, positive likelihood ratio of 4.18 (95% CI 2.4–7.3), negative likelihood ratio of 0.27 (95% CI 0.08–0.9) for CI-AKI prediction (Fig. 2).

**Fig. 3:**
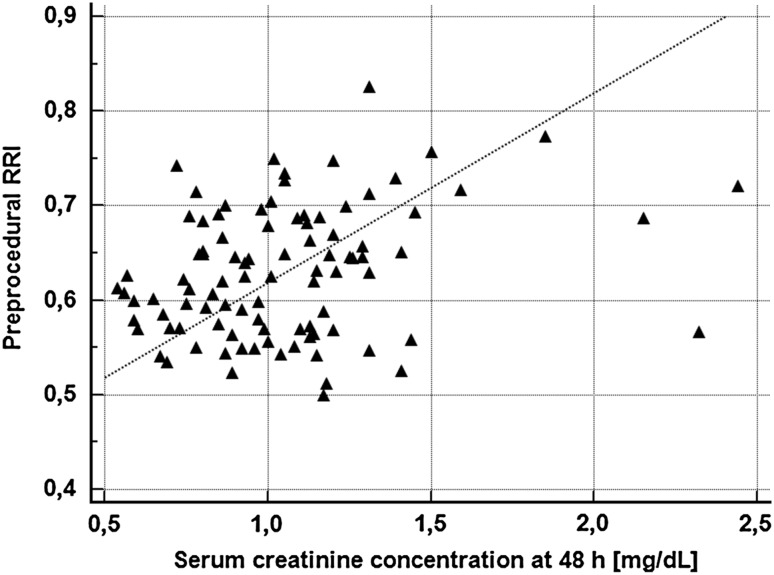
Correlation between pre-procedural renal resistive index and serum creatinine concentration at 48 h

The analysis of correlation revealed that both pre-procedural RRI (r = 0.24, p = 0.02; Fig. 3) and RPI (r = 0.20, p = 0.0497) positively correlated with SCr at 48 h, but not with the baseline SCr (RRI: r = 0.15, p = 0.14; RPI: r = 0.09, p = 0.36) and eGFR (RRI: r = −0.03, p = 0.72; RPI: r = 0.09, p = 0.34). Also, there was a statistical trend towards a positive correlation between RRI and SYNTAX score (r = 0.18, p = 0.07), and towards negative correlation between RRI and LVEF (r = −0.18, p = 0.07). Moreover, RRI positively corresponded with waist-to-hip ratio (r = 0.23, p = 0.02). SYNTAX score positively correlated with both total and weight-adjusted volume of CM (r = 0.58, p < 0.0001 and r = 0.58, p < 0.0001), as well as with IMT (r = 0.72, p < 0.0001) and EMT (r = 0.56, p < 0.0001).

## Discussion

This prospective study was designed to investigate into the pre- and intra-procedural risk factors of CI-AKI in subjects with stable angina or acute coronary syndromes, but without preexisting renal function impairment, who were referred for planned or urgent coronary angiography. Special consideration was given to baseline intra-renal blood flow parameters. The present study corroborated the significance of widespread vascular pathology in the prediction of CI-AKI in the setting of cardiology department. CAD severity reflected by SYNTAX score and the presence of PAD were found to be key predictors of CI-AKI in patients with preserved renal function, while renal vascular abnormalities further enhanced the predictive power of the model.

Our study constitutes, by far, the first report in literature showing successful application of RRI as a pre-procedural predictor of CI-AKI. According to present findings, baseline RRI and RPI values were significantly higher among subjects who exhibited CI-AKI following CA/PCI, in comparison to the rest of study participants. This inter-group difference was primarily conditioned by a trend towards lower intra-renal EDV among CI-AKI group. Although, both RRI and RPI constituted predictors of CI-AKI in univariate analysis, the multivariate model indicated that only RRI remained an independent predictor of CI-AKI onset from among all Doppler intra-renal flow indices. Similar findings, but in the setting of ICU, were obtained by Schnell and coworkers [13]. In this study, RRI assessed within 12 h after admission among ICU patients, was significantly higher in patients with stage 2 or 3 AKI, as compared to patients with AKI at stage 0 or 1 (RRI 0.80 vs. 0.66, p < 0.0001) [13]. Most importantly, RRI > 0.71 acquired within 12 h following admission was the only parameter predictive of AKI at stage 2 or 3 and outperformed serum and urinary cystatin C [13]. Correspondingly, our results support a similar predictive RRI threshold of 0.69, yet established before CM administration. High sensitivity of 78% and specificity of 81% for CI-AKI prediction at 48 h, suggest a possible application of RRI during initial check-up, e.g. concurrently with transthoracic echocardiography, for the risk stratification of CI-AKI onset.

The increased RRI value in our study was mainly conditioned by the tendency towards lower intra-renal EDV among patients who developed CI-AKI. We may speculate that greater RRI values reflect increased intra-renal vascular resistance related with endothelial dysfunction [8] or possibly renal arteriosclerosis. This baseline augmented intra-renal vascular resistance may act permissive to the tubular injury caused by highly concentrated viscous CM in renal outer medulla [18]. Contrast media cause an imbalance between vasodilative [19] and vasoconstrictive agents [20]. The vasoconstriction of afferent arteriole via adenosine triggered by contrast-induced overstimulation of tubuloglomerular feedback may also contribute to increased renal vascular resistance [21]. All these observations are line with the observed propensity towards increased RRI in subjects exhibiting CI-AKI. Last but not least, invasive measurement of intra-renal blood flow directly before and after CM administration revealed a significant increase of resistance index (defined as ratio of mean blood pressure and average peak renal velocity) and decrease of peak systolic velocity [7].

It is vital to note that, apart from renal vascular resistance, RRI is determined by several other parameters, including pulse and mean arterial pressure [22], heart rate and rhythm [23], blood oxygen saturation [24], renal vascular compliance and renal interstitial fibrosis [16]. Still, almost all these parameters might individually be associated with the risk of CI-AKI development. In the study by Huang et al. based on 448 patients subject to PCI, elevated baseline central pulse pressure was an independent predictor of CI-AKI [9]. Our results did not reveal significant correlation between RRI and pulse, nor systolic or diastolic blood pressure or heart rate, which may be related to the exclusion of extremely high pulse pressure variables and heart rate, along with the phenomenon of renal blood flow auto-regulation. Therefore, our findings may suggest that RRI delivers additional predictive value on top of the traditional characteristics of arterial stiffness.

Moreover, logistic regression analysis revealed that CI-AKI was accurately predicted by morphology of coronary lesions reflected by SYNTAX score, as well as the presence of PAD and DM/IFG/IGT and advanced age. These results underline the significance of vascular pathology and partially comply with former high-volume studies concerning risk factors of CI-AKI [2, 3, 6]. The significance of both SYNTAX score and PAD is probably related with the concomitant renal vascular pathology in patients with more advanced atherosclerosis, as well as higher requirement for CM (challenging vascular access site/longer duration of the procedure).

Also, coronary anomaly, referral for CABG, IMT and EMT value, although not included in the model, were associated with higher risk of renal function worsening based on univariate model (Table 5).

The final predictive model was characterized by a very good diagnostic power for CI-AKI prediction (Fig. 1). The main difference from previous reports was the lack of baseline kidney function and volume of CM in the final model. Still, weight-adjusted total volume of CM was associated with CI-AKI onset based on univariate model (OR = 1.99, p = 0.022) and was probably excluded from the model because of high collinearity with SYNTAX score. Baseline GFR and creatinine concentration did not predict CI-AKI onset in the described population. This may be explained by the applied exclusion criteria, as the study was designed to select patients with relatively good preliminary kidney function (mean eGFR of 80.7 mL/min/1.73 m^2^). Only 17 patients were characterized by eGFR between 50 and 60 mL/min/1.73 m^2^. Interestingly, these participants had baseline RRI values comparable with the rest of population (p = 0.67), suggesting that RRI is rather an indicator of susceptibility to AKI and contrast-mediated renal injury than a marker of impaired kidney function [25].

The results of logistic regression analysis allowed for the identification of patients, who could benefit the most from the pre-procedural screening with renal Doppler ultrasound. Analysis of RRI yields best results in terms of CI-AKI assessment in elderly patients with advanced peripheral and coronary atherosclerosis and presence of DM type 2. Based on the results of the study RRI assessment cannot be routinely recommended, yet it is worth consideration in subjects fraught with cardiovascular risk factors, in order to select individuals requiring more intensive peri-procedural hydration regimen with forced diuresis (furosemide preceded by volume expansion) [26], the cessation of possibly nephrotoxic drug (e.g. angiotensin converting enzyme inhibitors) [27], statin loading dose prior to procedure [28], radical limitation of contrast agent dose, as well as careful and prolonged post-procedural renal function monitoring (e.g. SCr assessment >72 h after the procedure).

The main limitation of the study is the imperfection of the renal Doppler ultrasonography itself, as patients with morbid obesity, extreme parameters of pulse pressure and heart rate and renal pathology had to be excluded from the study. Since this method is characterized by rather low repeatability, the RRI and RPI could have varied depending on the current view angle or patient’s position. Nevertheless, we have made an effort to overcome this shortcoming by way of using an arithmetic mean of serial measurements in two kidneys. During both pre- and post-procedural examination patients remained in supine position, as the majority of the patients were confined to this position following CA via femoral access. The ideal method should employ the measurement of resistive index by an invasive Doppler flow wire [7], which regrettably was not implemented in our study. Still, this technique may prolong the duration of the procedure and increase CM use and could trigger renal circulatory complications. Current measurement of RRI should not be applied to high-risk populations with severely impaired renal function, as the manuscript described predominantly low-to-medium CI-AKI risk patients. Due to the fact that last SCr was assessed at 48 h after the procedure, the rate of CI-AKI might have been underestimated.

## Conclusions

In the broader perspective, the results of the study underscore the importance of coronary, peripheral and renal vascular pathology in the development of CI-AKI in patients without preexisting renal pathology who are referred for coronary angiography. Pre-procedural evaluation of RRI is a relatively plain diagnostic tool, which independently contributes to risk stratification of CI-AKI.
